# Vaginal Cuff Closure in Minimally Invasive Hysterectomy: A Review of Training, Techniques, and Materials

**DOI:** 10.7759/cureus.1766

**Published:** 2017-10-11

**Authors:** Katherine Smith, Aileen Caceres

**Affiliations:** 1 Medical Education, UCF College of Medicine; 2 Minimally Invasive Gynecology, UCF College of Medicine

**Keywords:** minimally invasive surgery, laparoscopy, laparoscopic hysterectomy, vaginal cuff, hysterectomy, robotic surgery, barbed suture, knotless suture, vaginal cuff dehiscence, single site surgery

## Abstract

Hysterectomy is one of the most common surgeries performed each year and can be indicated for many gynecologic conditions. The development of minimally invasive surgery has transformed this procedure, resulting in improved outcomes, superior cosmesis, and quicker return to normal function. Vaginal cuff closure is a critical component of hysterectomy, with many variations in surgical technique and materials. This review provides an overview of intracorporeal suturing and knot-tying techniques at the level of a junior resident in obstetrics and gynecology and describes several validated models that have been developed to test resident skill level in vaginal cuff closure. We also provide a review of the literature regarding vaginal cuff closure techniques and suture materials, including knotless barbed sutures. Finally, a brief discussion of single-site surgery, the latest development in minimally invasive hysterectomy, will be provided. We hope to provide a better understanding of vaginal cuff closure for residents in the field of obstetrics and gynecology.

## Introduction and background

Hysterectomy is one of the most common surgeries performed on women in the United States, with approximately 600,000 performed each year [1-2]. The most common benign indications for hysterectomy include uterine leiomyomas, adenomyosis, abnormal uterine bleeding, endometriosis, and uterine prolapse. Hysterectomy is also performed for gynecologic cancers, including uterine, ovarian, fallopian tubal, cervical, and peritoneal cancers [1].

The first successful hysterectomies were performed in the nineteenth century using vaginal or abdominal incisions [3]. The abdominal and vaginal approaches to hysterectomy remained the only surgical options until 1989, when Reich performed the first laparoscopic hysterectomy [4]. Now, several variations of laparoscopic hysterectomy are available, including robot-assisted laparoscopic hysterectomy, laparoendoscopic single-site surgery (LESS), and natural orifice transluminal endoscopic surgery (NOTES). According to 2009 United States surveillance data, the surgical approaches to hysterectomy for benign disease were distributed as follows: 56 percent abdominal, 20 percent laparoscopic, 19 percent vaginal, and 5 percent robotic [5]. The minimally invasive approach to hysterectomy has been associated with improved outcomes when compared to the abdominal approach, including decreased morbidity, shorter hospital stay, and quicker return to normal activities [6]. These improved outcomes have contributed to the rising popularity of minimally invasive hysterectomy.

In minimally invasive hysterectomy, the vaginal cuff is sutured closed by a variety of possible techniques. Institutional and individual variation exists between the following elements of cuff closure: intracorporeal versus transvaginal closure, suture material, and suture technique. Significant evidence suggests that the rate of vaginal cuff dehiscence (VCD) is higher with intracorporeal closure of the cuff [7-9]. However, laparoscopic intracorporeal closure has been associated with longer postoperative vaginal length and shorter operative time [10]. The choice between laparoscopic and transvaginal sutures has been thoroughly studied and is outside the scope of this review.

We will provide an overview of commonly used intracorporeal suturing techniques in addition to training models that have been constructed for laparoscopic and robotic vaginal cuff closure techniques. We will also evaluate literature related to suture materials and techniques used for vaginal cuff closure in minimally invasive hysterectomy. Finally, we will discuss vaginal cuff closure techniques for single-site hysterectomy, one of the newest advancements in minimally invasive gynecologic surgery. This review should serve as an overview of vaginal cuff closure for trainees in the early years of an obstetrics and gynecology residency.

## Review

Laparoscopic suturing techniques

Intracorporeal suturing with laparoscopy is an essential skill for minimally invasive gynecologic surgery. There are many techniques for laparoscopic suturing; we will provide an overview of the techniques for placing interrupted sutures, running sutures, and figure-of-eight sutures. Additionally, we will discuss techniques used for intracorporeal knot tying.

When intracorporeal suturing is necessary, the needle is first introduced into the pelvis with the needle holder grasping the suture two to three centimeters from the needle. The needle is then held in place by the grasper so that it can be repositioned on the needle holder, approximately two-thirds distance away from the tip of the needle with an angle of 90° between the needle and the needle driver. The grasper then releases the needle. The needle is driven through the tissue at a 90° angle and rotated clockwise. The grasper then picks up the needle as it exits the tissue. A knot is tied intracorporeally after this first stitch. If an interrupted suture is desired, then the suture tails are cut. For a continuous suture, the needle is re-introduced through the tissue at a distance of approximately one centimeter from the first bite and the process continues until reaching the apex of the wound. A second knot is tied at the end of the suture line. Bite depth and tissue handling will be discussed in further detail later.

A figure-of-eight suture is used to close wounds that are under a high degree of tension and are often used as reinforcing sutures to strengthen wounds closed with a continuous running suture. Figure-of-eight sutures can be placed laparoscopically using the same basic principles as with traditional suturing by hand. The needle is introduced into the tissue at a 90° angle and driven clockwise through the tissue so that the needle emerges at a point equidistant from the incision as the point of needle entry. Without tying a knot, the needle is then driven into the tissue again in a bite on the same side of the incision as the first point of needle entry, approximately one centimeter away. This second bite is driven through the tissue so that the needle emerges adjacent to the point of exit of the first bite. The suture should now resemble a square with an “X” connecting the angles. At this point, a knot is tied to secure the figure-of-eight suture.

Intracorporeal knot-tying (ICKT) is regarded as the most technically difficult laparoscopic skill. Even for expert laparoscopists, ICKT can be challenging and time-consuming [11]. Regardless of difficulty, these knots must have equal strength to hand-tied knots to prevent complications. There are several techniques that are used for ICKT as well as tools that can be used as a substitute for knot-tying, including the Lapra-Ty clip (Ethicon Endosurgery, Cincinnati, Ohio) and barbed sutures.

**Figure 1:**
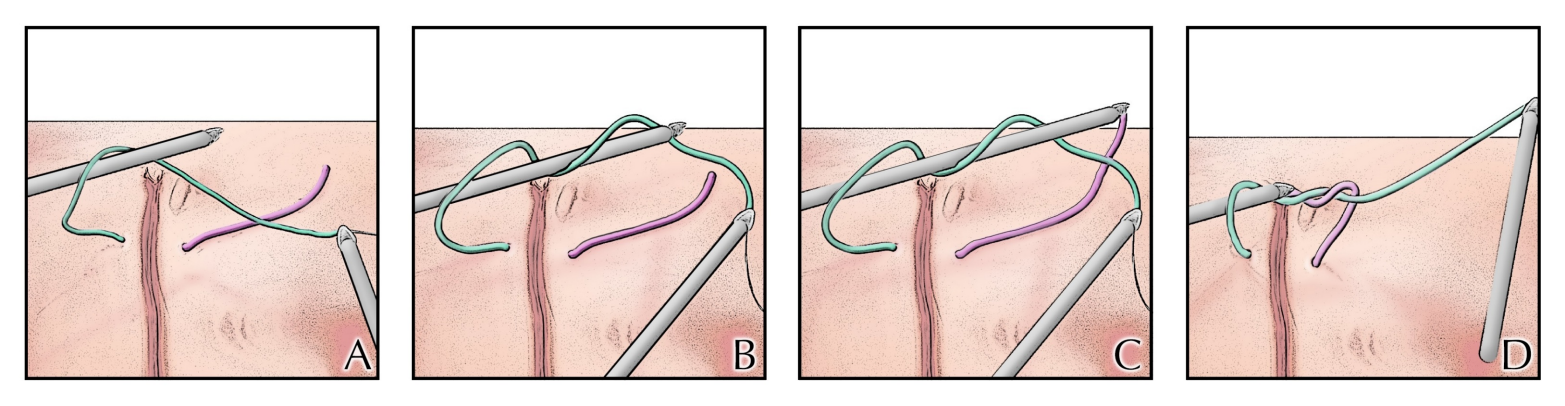
Looped technique of intracorporeal suturing The suture is grasped by the right needle driver. The right needle driver is then used to make two loops over the left needle driver (Panels A & B). The left needle driver then grasps the tail of the suture (Panel C). The knot is then secured (Panel D). To square the knot, this loop is followed by a single reverse loop and then one additional forward loop. Image Credit: Ryan Dickerson, University of Central Florida, 2017.

**Figure 2:**
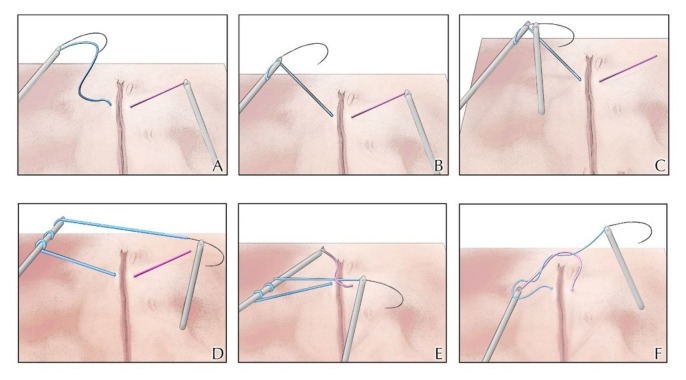
Coiled technique for intracorporeal suturing The left needle driver grasps the suture distal to the needle (Panel A). The left needle driver is then rotated to wind the suture around its axis (Panel B). The right needle driver then grasps the needle and maintains the wind on the left needle driver (Panels C & D). Finally, the left needle driver grasps the tail end of the suture (Panel E) and secures the first throw (Panel F). To square the knot, the following throw is performed with a reverse wind and then a throw with a forward wind. Image Credit: Ryan Dickerson, University of Central Florida, 2017.

Similar to hand-tied knots, a square surgeon’s knot is the ideal intracorporeal knot to approximate tissues under tension. Using two instruments, a surgeon’s knot can be created by either a “loop” or a “winding” technique. The more traditional loop technique is accomplished by using the dominant needle driver to make two loops over the non-dominant needle driver. The non-dominant needle driver then grasps the tail of the suture. To square the knot, this loop is followed by a single reverse loop and then one additional forward loop (Figure 1). In the winding method, the needle is first released from the driver. Then, the non-dominant needle driver grasps the suture distal to the needle. The non-dominant driver is then rotated to wind the suture around its axis. The dominant needle driver then grasps the needle and maintains the wind on the non-dominant needle driver. Finally, the non-dominant needle driver grasps the tail end of the suture and secures the first throw. The following throw is performed with a reverse wind and then a throw with a forward wind (Figure 2) [12].

**Figure 3:**
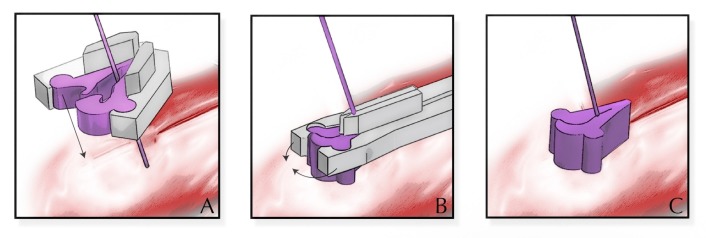
Depiction of the Lapra-Ty clip (Ethicon Endosurgery, Cincinnati, Ohio) Traction is placed on the suture while the open clip is guided to the tissue (Panel A). The clip is then deployed flush with the tissue (Panel B). After releasing the traction, the clip dimples into the tissue (Panel C). Image Credit: Ryan Dickerson, University of Central Florida, 2017.

To improve efficiency, many laparoscopic surgeons use a Lapra-Ty clip as a substitute for knot tying. When using a Lapra-Ty clip, the surgeon places traction on the suture and applies the clip flush with the tissue. After releasing the traction, the Lapra-Ty dimples into the tissue according to the degree of traction (Figure 3). Although using the Lapra-Ty clip is technically less challenging than tying an intracorporeal knot, it involves the introduction of a foreign device to the vaginal cuff. If the patient develops a hematoma or seroma, there is a possibility that the clip could harbor an infection. Other disadvantages with the Lapra-Ty clip include increased material cost and the possibility of slippage. Lapra-Ty slippage could result in a loosening of the suture, inadequate tissue reapproximation, and potential wound dehiscence. The optimal suture types and sizes to use with the Lapra-Ty clip to maximize holding strength and minimize slippage include 2-0 and 3-0 Vicryl (Ethicon Endosurgery, Somerville, New Jersey), 2-0 Monocryl (Ethicon Endosurgery, Somerville, New Jersey), and 2-0 polydioxanone suture (PDS) (Ethicon Endosurgery, Somerville, New Jersey) [13].

Barbed sutures are another substitute for knot-tying that has gained increasing popularity, particularly in gynecologic surgery. The utilization of barbed sutures in laparoscopic hysterectomy will be covered in greater detail in the following sections.

Intracorporeal knot-tying is an essential skill for the laparoscopic surgeon, but extracorporeal knot-tying using a knot pusher should be an additional component of the surgeon's toolbox. To tie an extracorporeal knot, a stitch must be placed in the tissue such that both ends of the suture are external to the abdomen. A knot is tied extracorporeally and then a knot pusher is mounted adjacent to the knot along the suture line. Traction is placed on both ends of the suture while the knot pusher guides the knot toward the tissue. The knot pusher is then released from the tissue, and these steps are repeated for additional throws.

Although proper technique is a vital element of suturing and knot-tying, tissue handling is an equally critical element of vaginal cuff closure. When suturing the vaginal mucosa, it is important to grab adequate bites of tissue at the serosal layer. Bites that are too shallow may leave edges of the cuff that bleed. Additionally, individual bites should be taken for each half of the stitch so that the needle is visible in the middle of the incision for each bite. This technique ensures that the suture is in the appropriate layer of tissue.

Training models for laparoscopic suturing of the vaginal cuff

Several models have been developed and validated for both practicing and assessing the surgical skills required for laparoscopic suturing of the vaginal cuff (Table 1). The first such model was developed by Arden et al. in 2008 [14]. In this study, the researchers developed a box model (Pelv-Sim) for training residents in several laparoscopic skills specific to gynecologic surgery, including closure of the vaginal cuff, transposition of an ovary to the pelvic side wall, ligation of an infundibulopelvic ligament, and closure of a port-site fascial incision. Another model for laparoscopic vaginal cuff closure was reported by Weizman et al. in 2015 [15]. The model constructed in this study consisted of a latex vaginal cuff model in the fundamentals of laparoscopic surgery (FLS) box trainer and was validated in a study of five novice and five expert surgeons. King et al. described a reusable, affordable plastic box model for practicing laparoscopic vaginal cuff closure; this model utilized a real camera and tower, thus offering participants a more realistic experience [16]. Tunitsky-Bitton et al. developed a vaginal cuff closure simulation model by adapting the FLS box model [17]. The advantage of this model is that the FLS model is readily available in many teaching institutions, thus allowing trainees to practice this technique independently. Training tools for residents to practice laparoscopic suturing skills have become especially important due to the recent shift to robotic surgery in lieu of laparoscopy. These models allow residents to practice laparoscopic suturing techniques despite exposure to fewer purely laparoscopic cases during their training.

In addition to laparoscopic models, robotic models have been developed for closure of the vaginal cuff. Finan et al. developed a dry lab to replicate several tasks performed in robotic hysterectomy, including a dexterity exercise, bladder flap development, sealing and cutting the round and infundibulopelvic ligaments, skeletonizing the uterine vessels, and suturing the vaginal cuff. This model utilized a “beer coozie” vaginal cuff placed within a box trainer for the da Vinci robotic platform (Intuitive Surgical, Sunnyvale, California) with a single operative console and a touch-screen monitor for attendings to instruct residents [18]. Kiely et al. used the robotic model developed by Finan et al. to evaluate trainee improvement in the robotic surgical technique with the use of the simulator. They found that the robotic training resulted in an improved ability to perform inanimate tasks robotically, but the translation from virtual reality to inanimate tasks was incomplete. Additionally, the training program provided the greatest benefit to trainees with the least robotic surgery experience [19].

**Table 1:** Training models constructed for laparoscopic and robotic vaginal cuff closure skill development

Author	Technique	Participant Characteristics	Description of Model	Tasks Assessed
Arden et al. (2008) [14]	Laparoscopic	19 obstetrics & gynecology residents 10 medical students rotating on obstetrics & gynecology service	Pelv-Sim box model trainer with two ports for laparoscopic instruments, a simulated open vaginal cuff constructed from burlap, an ovary, two infundibulopelvic (IP) ligaments, and fascia with optional attachments for the laparoscope and video tower.	1) Vaginal cuff closure 2) Transposition of an ovary to the pelvic side wall 3) Ligation of an infundibulopelvic ligament 4) Closure of a port-site fascial incision
Weizman et al. (2015) [15]	Laparoscopic	5 "experts" (fellows or senior surgeons) 5 "novices" (medical students)	Vaginal cuff model constructed from liquid latex and placed into the fundamentals of laparoscopic surgery (FLS) box trainer.	Vaginal cuff closure
King et al. (2015) [16]	Laparoscopic	5 "experts" (attending surgeons) 5 "advanced novices" (fellows) 15 "early novices" (residents)	Vaginal cuff model constructed from corduroy fabric (vagina) and an internal neoprene layer (vaginal mucosa) and placed in a box trainer with ipsilateral and suprapubic ports.	Vaginal cuff closure
Tunitsky-Bitton et al. (2016) [17]	Laparoscopic	19 "experts" (attending surgeons) 21 "trainees" (senior residents and fellows)	Repurposed uterine manipulator, sacrocolopexy tip/vaginal stent, and a vaginal cuff constructed from neoprene material and lined with swimsuit material placed into the FLS box trainer.	Vaginal cuff closure
Finan et al. (2010) [18]	Robotic	Obstetrics and gynecology residents	"Beer huggie" vaginal cuff model placed within the Intuitive da Vinci S model robot with a single operative console and a touch-screen monitor for attendings to instruct residents.	1) Dexterity exercise 2) Bladder flap development 3) Sealing and cutting the round and IP ligaments 4) Skeletonizing the uterine vessels 5) Vaginal cuff closure
Kiely et al. (2015) [19]	Robotic	13 in the trainee group (8 residents, 5 attendings) 10 in the control group (9 residents, 1 attending)	"Beer huggie" vaginal cuff model with balloons to represent the bladder and rectum and placed within a trainer for the da Vinci surgical system.	Vaginal cuff closure

The vaginal cuff models described by these studies provide promising tools for surgical training. Regardless of the model used, it is important that trainees have access to resources to practice their surgical skills, especially for a skill as critical as closure of the vaginal cuff. 

Barbed versus standard suture

The barbed suture was designed in the 1950s by Dr. John Alcamo but was not widely used or manufactured until 2004, when the Quill Knotless Tissue Closure Device (Angiotech Pharmaceuticals, Vancouver, British Columbia) was approved by the United States Food and Drug Administration. Now, the three commercially available barbed sutures include the V-Loc Absorbable Wound Closure Device (Covidien Healthcare, Mansfield, Massachusetts), the Quill Self-Retaining System, and the Stratafix (Ethicon Endosurgery, Somerville, New Jersey) [20].

Barbed sutures are manufactured by slicing longitudinal “barbs” into straight sutures and then adding a looped closure or needle at each end. They can have a needle at one end (unidirectional barbed suture) or needles at both ends with a change in barb direction in the midline of the suture (bidirectional barbed suture) [21].

The benefit of barbed sutures primarily originates from their ability to reapproximate tissue without the use of surgical knots. The surgical literature recognizes that the weakest part of a suture line is at the knot, and tensile strength is reduced in the suture adjacent to the knot [22]. Additionally, the elimination of knot-tying can reduce operative time, even with an expert surgeon [23]. This is especially important in laparoscopic surgery, where tying surgical knots can be more challenging and time-consuming than in open cases. Finally, barbed sutures have demonstrated a greater load to failure than traditional sutures, defined as the force in Newtons required to cause suture breakage or tissue tearing [24]. This finding is likely related to the distribution of force over a larger surface area by the barbs.

In 2008, Greenberg and Einarsson were the first to report the use of barbed sutures in laparoscopic gynecologic surgery [25]. They described eight cases (five laparoscopic myomectomies and three total laparoscopic hysterectomies) where barbed suture had been used to close the uterus (myomectomy) or the vaginal cuff (hysterectomy). Einarsson et al. (2011) published the first large cohort study addressing barbed suture use in gynecology; they evaluated 138 laparoscopic myomectomies using bidirectional barbed sutures to facilitate the closure of a hysterostomy site [26]. They found a reduction in operative time and length of hospital stay without any significant changes in perioperative complications. These findings suggested that barbed sutures can be safely used in gynecologic surgery while offering benefit to the surgeon and the patient.

Since the introduction of barbed sutures, several studies have evaluated the utility of barbed sutures for vaginal cuff closure during minimally invasive hysterectomy. The most important considerations regarding the use of these materials for cuff closure are operative time, postoperative complications, and cost.

Eight of these studies compared the operative time between barbed suture cases and conventional suture cases (Table 2) [27-34]. On average, cases using a barbed suture to close the vaginal cuff showed a decreased total operative time by 15.6 minutes (136.9 minutes vs. 151.7 minutes), compared with cases using a conventional suture. Three studies evaluated vaginal cuff closure time independently; the barbed suture groups saw a mean decrease of 5.4 minutes [28,31,35].

**Table 2:** Operative time differences between barbed and conventional sutures in previous studies *extracorporeal knots; **intracorporeal knots

	Barbed Suture (min)	Conventional Suture (min)	Difference (min)	p-value
Total Operative Duration				
Nawfal et al. (2012) [27]	135	175	40	p < 0.001
Ardovino et al. (2013) [28]	131.5	133.4 * 141.5 **	1.9 * 10.0 **	not significant
Bassi et al. (2013) [29]	115.9	118.6	2.7	not significant
Bogliolo et al. (2013) [30]	122	136	6	p < 0.010
Song et al. (2014) [31]	92	105.2	13.2	p = 0.002
Medina et al. (2014) [32]	185	180.4	4.6	not significant
Zhou et al. (2014) [33]	220.2	272.8	52.8	p < 0.001
Cong et al. (2016) [34]	93.46	102.43	9	p = 0.002
mean	136.9	151.7	15.6	
Vaginal Cuff Closure Time				
Ardovino et al. (2013) [28]	3.9	6.2 * 7.1 **	2.3 * 3.2 **	p < 0.010
Song et al. (2014) [31]	11.4	22.5	11.1	p < 0.001
Kim et al. (2016) [35]	12.2	7.2	5	p < 0.001
mean	9.2	10.8	5.4	

Postoperative outcomes are another concern when comparing suture materials for the vaginal cuff. Overall, previous studies indicate that there may be a modest decrease in intraoperative blood loss with barbed suture use, but most studies did not find this result to be significant. Similarly, several studies reported a decreased incidence of postoperative vaginal bleeding, vaginal cuff cellulitis, and postoperative hospital stay with barbed sutures than with conventional sutures, but most did not report a significant difference between groups (Table 3). However, Seidhoff et al. reported a significantly higher incidence of vaginal cuff cellulitis with barbed sutures [36]. Overall, the literature does not indicate a significant variation in the incidence of postoperative complications with suture choice.

Vaginal cuff dehiscence (VCD) is one of the most serious complications related to the vaginal cuff, thus, was evaluated by all the studies. Seidhoff et al. and Rettenmaier et al. both reported a significantly decreased rate of dehiscence with barbed sutures [36-37]. However, many of the studies did not find a significant difference between groups. Because VCD is an exceedingly rare complication, it is difficult to demonstrate a decrease in the incidence of this event. The heterogeneity of dehiscence rates and the overwhelming proportion of non-significant results in the published literature suggest that the rate of VCD is either minimally changed or unchanged using barbed sutures in place of standard suture materials. Outcomes for the barbed suture studies can be found in Table 3 [27-39].

**Table 3:** Postoperative outcomes evaluated by studies comparing barbed sutures and conventional sutures for vaginal cuff closure Effects are reported as the barbed suture group relative to the control group of conventional suture materials unless otherwise specified (i.e. “decreased” represents a lower reported incidence in the barbed suture group). The O symbol denotes that the endpoint was not evaluated in the study. *Bassi et al. also reported a higher incidence of postoperative fever in the barbed suture group (p=0.003) [29]. ‡p-value not reported. †reported as 4.2% of 229 cases with conventional sutures. RCT = randomized controlled trial.

Author	Type of Study	Number of Cases	Intraoperative Blood Loss	Postoperative Vaginal Bleeding	Vaginal Cuff Cellulitis	Vaginal Cuff Dehiscence	Length of Stay	Surgical Difficulty
Bogliolo et al. (2013) [30]	retrospective	88	o	no significant difference	o	none	o	o
Cong et al. (2016) [34]	retrospective	490	decreased by 9 ml (p=0.019)	o	o	none	decreased by 1.6 days (p=0.000)	o
Einarsson et al. (2013) [38]	RCT	63	o	no significant difference	o	1 with barbed suture, 1 with conventional suture‡	o	o
Kim et al. (2016) [35]	retrospective	170	o	no significant difference	o	none	o	o
Medina et al. (2014) [32]	retrospective	232	no significant difference	decreased (p=0.030)	o	4 cases with conventional suture, 1 case with barbed suture (p=0.600)	no significant difference	o
Nawfal et al. (2012) [27]	retrospective	202	decreased by 25 ml (p<0.001)	o	o	1 case with conventional suture (p=0.720)	less likely to stay longer than 1 day (p=0.006)	o
Neubauer et al. (2013) [39]	retrospective	134	o	no significant difference	no significant difference	none	o	o
Rettenmaier et al. (2015) [37]	retrospective	1876	o	o	o	14 cases with conventional suture (p=0.034)	o	o
Seidhoff et al. (2011) [36]	retrospective	387	o	decreased (p=0.008)	decreased with conventional suture (p=0.030)	9 cases with conventional suture (p=0.008)†	o	o
Zhou et al. (2014) [33]	retrospective	93	decreased by 110 ml (p=0.020)	no significant difference	no significant difference	1 with barbed suture (p>0.05)	no significant difference	o
Ardovino et al. (2013) [28]	RCT	61	no significant difference	no significant difference	o	none	o	decreased (p<0.010)
Bassi et al. (2013) [29] *	retrospective	202	no significant difference	no significant difference	o	none	no significant difference	o
Song et al. (2014) [31]	case-control	102	no significant difference	o	o	none	no significant difference	decreased (p<0.001)

Although peri-operative outcomes appear similar for barbed and traditional sutures, other variables should be weighed when choosing materials. Importantly, cost should be a consideration. Barbed sutures have a markedly higher material cost than traditional sutures. However, the reduced operative time with barbed sutures necessitates a consideration of the cost of operating room time. In 2014, Zeybek et al. analyzed the costs of laparoscopic and robotic operating room time for a hysterectomy. Based on their data, they concluded that laparoscopic operating room time costs $4,961 for the first 30 minutes and $2,426 for each additional 30 minutes. For robotic operating room time, the cost was $5,513 for the first 30 minutes and $2,756 for each additional 30 minutes [40]. Although these data only describe the experience of one institution, it can be reasonably concluded that decreased operating room time would result in a significantly reduced overall cost for minimally invasive hysterectomy. Thus, the reduced operative time with barbed sutures may offset the higher material costs.

Overall, the literature suggests that barbed sutures are a useful tool for vaginal cuff closure. The literature shows that these materials are non-inferior to traditional sutures, and some studies suggest a modest benefit in postoperative complications and operative duration. Despite their high material cost, reduced operating room time may offset the higher price for barbed sutures. Large clinical trials are recommended to demonstrate a definitive benefit with barbed sutures. 

Suture technique

When compared to suture materials, the choice of closure technique for the vaginal cuff is a less-studied element of minimally invasive hysterectomy. The cuff closure technique varies among surgeons and is often chosen based on surgeon comfort and experience. In a systematic literature review, Uccella et al. suggested that the vaginal cuff suture technique may play a role in the rate of postoperative vaginal cuff dehiscence [7]. However, the ideal technique remains elusive. A large retrospective cohort study of 1924 patients published by O’Hanlan et al. evaluated a standardized laparoscopic vaginal cuff closure technique. Their technique incorporated the placement of sutures five millimeters deep from the vaginal edge with five millimeters between sutures, incorporation of the uterosacral ligaments with the pubocervical fascia at the angles of the wound, and suturing the bladder peritoneum over the vaginal cuff edge. This cohort experienced a low rate of vaginal cuff complications of 2.29%, and only 0.99% of patients required reoperation for a complication of the vaginal cuff, thus indicating that their methods are associated with few vaginal cuff complications [41]. Although these results are informative, there remains a deficiency of prospective trials comparing suture techniques. In our literature search, we identified only three studies that evaluated different intracorporeal suture techniques of the vaginal cuff in minimally invasive hysterectomy (Table 4) [42-44].

**Table 4:** Suture techniques and outcomes for previous studies comparing vaginal cuff closure techniques RCT=randomized controlled trial. VCD=vaginal cuff dehiscence. Vicryl (Ethicon Endosurgery, Somerville, New Jersey); Lapra-Ty (Ethicon Endosurgery, Cincinnati, Ohio); V-Loc (Covidien Healthcare, Mansfield, Massachusetts).

Author	Type of Study	Number of Cases	Study Groups	Outcomes	Findings
Blikkendaal et al. (2012) [42]	retrospective	331	1) transvaginal interrupted 2) laparoscopic interrupted 3) laparoscopic single-layer running a. bidirectional barbed suture b. running Vicryl suture	vaginal cuff dehiscence	Eight VCDs (p=0.707): - 1 after transvaginal interrupted - 3 after laparoscopic interrupted - 4 after laparoscopic running
Landeen et al. (2016) [43]	RCT	263	1) single-layer continuous sutures 2) single-layer continuous sutures with three reinforcing figure-of-eight sutures	vaginal cuff dehiscence operative time blood loss length of stay urinary tract infection postoperative pain	Four VCDs: - 3 in non-reinforced group - 1 in reinforced group * All VCDs were in current smokers. No significant difference for other outcomes.
Tsafrir et al. (2016) [44]	RCT	90	1) running 2.0 V-Loc 2) interrupted 0 Vicryl 3) running 0 Vicryl with Lapra-Ty	vaginal cuff dehiscence vaginal cuff closure time peri-operative bleeding continuous pain dyspareunia vaginal bleeding vaginal discharge	No cases of vaginal cuff dehiscence. Significant difference in long-term vaginal pain (p=0.01): - 3 patients in Group 3 (running 0 Vicryl with Lapra-Ty) - 0 patients in the other groups No significant difference for other outcomes.

Blikkendaal et al. found no significant difference between the rates of VCD in a retrospective study of 331 cases of vaginal cuff closure by either transvaginal interrupted sutures, laparoscopic interrupted sutures, or laparoscopic single-layer running sutures [42]. Similarly, Tsafrir et al. experienced no cases of VCD in a randomized controlled trial of 90 cases of intracorporeal vaginal cuff closure; their analysis compared a running 2.0 barbed suture, an interrupted 0 Vicryl suture, and a running 0 Vicryl suture with Lapra-Ty [44]. Contrasting with these results, Landeen et al. identified a significant difference in VCD between two cuff closure techniques in a randomized controlled trial of 263 cases. Their evaluation compared cuff closure with a single-layer continuous running suture and a single-layer continuous running suture with three reinforcing figure-of-eight sutures [43]. There were four cases of VCD in their sample; three of the four VCDs were in the non-reinforced arm of the trial. All four VCDs were in current smokers, but the relative risk of VCD in smokers in the non-reinforced group was 4.23 times higher than of VCD in smokers of the reinforced suture group. These findings suggest that reinforcing sutures reduce the rate of dehiscence. The benefit of these technical refinements can be better demonstrated in future clinical trials using sufficient sample size to detect differences in outcomes.

Overall, these limited studies provide insufficient data to demonstrate the superiority of a single cuff closure technique. Indeed, there are likely many acceptable techniques to close the vaginal cuff; an ideal technique may never be identified and likely varies based on the surgeon and the clinical situation. Regardless, the current paucity of the literature regarding the vaginal cuff closure technique in minimally invasive hysterectomy necessitates the development of large trials with adequate power.

Single-site hysterectomy

Laparoendoscopic single-site surgery (LESS) is an advanced minimally invasive technique that has been recently introduced into clinical practice. This technique utilizes a single trocar for both optics and instrumentation, thus improving cosmetic outcomes and reducing complications related to the placement of ancillary trocars. LESS is a surgically challenging platform because of poor ergonomics and restricted space for instruments, thus increasing instrument collision and making it more difficult to obtain adequate triangulation. These restrictions make closure of the vaginal cuff particularly challenging. The ideal approach to vaginal cuff closure in robotic single-site hysterectomies is currently being refined, and few studies have evaluated the optimal approach for cuff closure. Many studies of single-site hysterectomies use barbed sutures for vaginal cuff closure because these materials eliminate the need for intracorporeal knot-tying, which is particularly demanding in single-site surgery due to restricted movement and reduced space for instruments. Shin and colleagues describe an interesting technique using barbed sutures for vaginal cuff closure in single-site hysterectomy. In a series of 100 cases, they used a V-Loc unidirectional barbed suture with a straightened needle. They concluded that using a straightened needle for cuff closure results in shorter operative time and reduced technical difficulty [45].

Although barbed sutures may be easier to use in single-site surgery, surgeons should develop skills for intracorporeal knot-tying for this platform. Akdemir et al. applied a standardized intracorporeal cuff suture technique to 24 cases of robotic single-site total hysterectomy using intracorporeal interrupted single stitches with a 0 Vicryl suture with a 40-mm ½-circle cutting needle [46]. Using this technique, the surgeons found that the learning curve for intracorporeal suturing of the vaginal cuff in LESS reached a plateau at around 14 procedures, with a reduction of the average vaginal cuff closure time from 26.4 minutes in the first 14 cases to 18.7 minutes in the following 10 cases. Escobar et al. also found similar results, with surgical proficiency reached at 10-15 cases [47]. However, Paek et al. reported a longer timeline before surgical proficiency is attained, at about 40 cases [48]. Regardless, these findings suggest that although the learning curve is steep for intracorporeal suturing of the vaginal cuff in LESS, it can be overcome with sufficient practice.

The utilization of LESS in gynecologic surgery is relatively new, with limited outcome data available. Yang et al. published a systematic review of six randomized controlled trials and 12 retrospective studies, including a total of 3,725 patients; their analysis shows that, compared to multiport laparoscopic hysterectomy, single-site laparoscopic hysterectomy had a higher procedure failure rate, longer operative time, shorter length of stay, and faster return to bowel activity [49]. The procedure failure rate was mostly due to the requirement for extra ports, which would presumably be reduced with additional surgeon experience. Similarly, increased operative time in single-site surgery could be related to surgeon experience and may be reduced after the surgeon is exposed to more single-site cases. Notably, vaginal cuff closure is one of the most time-consuming steps in single-site hysterectomy. Postoperative outcomes were similar between single-site and multi-port hysterectomies, including postoperative pain and serious complications such as vaginal cuff dehiscence. Although outcomes are similar between these techniques, single-port surgery has been associated with greater cosmetic satisfaction compared to two-port or four-port procedures [50].

Single-site surgery is still in its infancy in the field of gynecology. The utilization of this technique for hysterectomy poses considerable technical challenges to the surgeon, especially during vaginal cuff closure. These challenges can result in increased operative duration, but the literature suggests that these hurdles can be overcome with sufficient experience. Similar postoperative outcomes between single-site and multi-port hysterectomy combined with improved cosmesis make LESS a viable alternative for traditional laparoscopic or robotic hysterectomy, especially for younger, non-obese patients in good physical condition. There is a paucity of studies on optimal technique for vaginal cuff closure in single-site hysterectomy. Further trials are warranted to refine the approach to intracorporeal suturing of the vaginal cuff, one of the most challenging components of single-site hysterectomy.

## Conclusions

Vaginal cuff closure is a component of minimally invasive hysterectomy with significant variance in technique and suture choice between surgeons and institutions. Transvaginal suturing has been touted as a superior technique to laparoscopic sutures due to historically lower rates of vaginal cuff dehiscence. However, new techniques and materials have been introduced that may improve the strength of laparoscopic and robotic closure. Since the introduction of barbed sutures, they have been shown to have utility in gynecologic surgery. Several studies suggest that a barbed suture may be superior to a conventional suture in postoperative outcomes and operative time, although the benefits are modest. The increased cost of barbed sutures may be offset by reduced time in the operating room. Suture technique is a less-studied variable in vaginal cuff closure. The literature suggests that technical refinements, specifically the addition of reinforcing sutures, may provide benefit in postoperative outcomes. However, prospective trials with a large sample size are necessary to identify a superior technique. The recent introduction of laparoendoscopic single-site surgery to the field of gynecology provides the possibility of further advancements in minimally invasive hysterectomy. The best technique for vaginal cuff closure in single-site surgery has not yet been established, as this is a cutting-edge field with few published trials. We believe that with new technologies and increased surgeon experience in minimally invasive techniques, intracorporeal vaginal cuff closure in minimally invasive hysterectomy will no longer be viewed as higher risk than transvaginal cuff closure.
